# Assessing Food Liking: Comparison of Food Liking Questionnaires and Direct Food Tasting in Two Cultures

**DOI:** 10.3390/nu10121957

**Published:** 2018-12-11

**Authors:** Uracha Wanich, Dhoungsiri Sayompark, Lynn Riddell, Sara Cicerale, Djin Gie Liem, Mohammadreza Mohebbi, Susie Macfarlane, Russell Keast

**Affiliations:** 1 Centre for Advanced Sensory Sciences, School of Exercise and Nutrition Sciences, Deakin University, 1 Gheringhap Street, Geelong 3220, Australia; u.wanich@deakin.edu.au (U.W.); sara.cicerale@deakin.edu.au (S.C.); gie.liem@deakin.edu.au (D.G.L.); 2 Faculty of Science and Technology, Rajamangala University of Technology Tawan-ok, Chonburi 20110, Thailand; deakinds@gmail.com; 3 Institute for Nutrition and Physical Activity Research, School of Exercise and Nutrition Sciences, Deakin University, 1 Gheringhap Street, Geelong 3220, Australia; lynn.riddell@deakin.edu.au; 4 Biostatistics Unit, Faculty of Health, Deakin University, 1 Gheringhap Street, Geelong 3220, Australia; m.mohebbi@deakin.edu.au; 5 Deakin Learning Futures, Health Pod, Deakin University, 1 Gheringhap Street, Geelong 3220, Australia; susie.macfarlane@deakin.edu.au

**Keywords:** cross-cultural, food liking, sensory, questionnaire, hedonic

## Abstract

Food liking can be directly measured in specialised sensory testing facilities; however, this method is not feasible for large population samples. The aim of the study was to compare a Food Liking Questionnaire (FLQ) against lab-based sensory testing in two countries. The study was conducted with 70 Australian and Thai participants (35 Australian, 35 Thai, mean (SD) age 19 (3.01) years, 51% men). Participants completed a FLQ (consisting of 73 food items Australia, 89 Thai) and then, after tasting the food, rated their liking of a selection of 10 commercially available food items using a nine-point hedonic scale. Both tasks were completed on the same day and were repeated one week later. The reliability of and a comparison between methods was determined using Intra-Class Correlation Coefficients (ICC), and the difference was assessed using an independent sample *t*-test. The results indicate that the test-retest reliability of FLQ and the laboratory-based liking assessment range was moderate (0.40–0.59) to excellent (0.75–1.00). There were significant differences for the FLQ and the laboratory-based liking assessment between countries for three food items: soft drink, instant vegetable soup, and broccoli (*p* < 0.01). However, the data produced from the FLQ reflects the laboratory-based liking assessment. Therefore, it provides representative liking data in large population-based studies including cross-cultural studies.

## 1. Introduction

Obesity represents the largest preventable disease worldwide and is a contributor to ill-health outcomes including cardiovascular disease, stroke, type 2 diabetes, hypertension, arthritis, respiratory disorders, and certain cancers [1]. Whilst the causes of obesity are multi-factorial and complex, they are embedded within energy imbalances brought about by psychological, cultural, personal, environmental, lifestyle, and dietary factors which favour excessive energy intake coupled with sedentary behaviour [2]. Energy imbalances due to overconsumption of food are common, especially, given discretionary foods high in palatable fat, sugar, and salt are increasing in abundance in both developed and developing nations [3,4].

An individual’s response to food is multi-dimensional and dynamic. Environment, experience, and physical state are all factors that may influence liking decisions at any point in time [5]. It is the liking or prospective liking of a food that is one of the key drivers of consumption [5,6]. The impact of taste and food preference on food intake is also influenced by age and sex and can be modified by distorted eating behaviours [6,7,8,9].

In adults, food flavour has an important influence on food choice [10]. Liking a food’s flavour is an important driver of short-term food consumption, as those adults who enjoy the food they are consuming tend to eat more of it [5]. This can result in health issues related to the overconsumption of food [5]. For example, an individual’s flavour preferences can have an effect on disease risk by influencing food consumption, particularly the consumption of foods high in fat, sugar, and salt. A study by Duffy et al. [11] demonstrated that the liking of fatty foods was positively correlated with fat intake and the liking for fibre-rich foods was positively correlated with fibre intake. Further, a positive relationship between the liking for fatty foods, body weight, and systolic blood pressure was found. This relationship between food liking and dietary intake was also observed in a large study by Mejean et al. Those with a higher liking for fatty foods had an increased intake of total energy, fat, and certain foods (high in fat) such as meat, butter, desserts, and pastries [12] and a positive relationship between the liking for fatty foods and obesity risk was observed.

There is a paucity of research comparing Food Liking Questionnaires (FLQ) and laboratory-based food acceptance. Cardello and Maller [13] examined the relationship between FLQ and laboratory acceptance testing both using a nine-point hedonic scale with the authors observing higher ratings in the laboratory acceptance testing compared with the FLQ. The authors also found positive, but mostly weak to moderate, correlations between the two methods. In addition, FLQ can be used to explore the relationship between food liking and food consumption. For example, Duffy et al. [14] used a questionnaire to determine food preference and intake to predict the dietary determinants of cardiovascular disease risk factors in 422 US male adults. This study showed that the preference for fatty food, intake of low-fibre food, and alcohol was associated with cardiovascular disease risk factors. Carbonneau et al. [15] developed and validated a food liking questionnaire which aimed to predict the influence of food liking on food and energy intake. A significant correlation was observed between liking scores in the FLQ and self-reported frequency of food consumption (r = 0.19–0.39, *p* < 0.05). French et al. [16] have used a self-report measure of the liking and wanting of high-fat food (among other measures such as the three-factor eating questionnaire) to investigate the association with energy intake and individual differences in eating behaviours. This study demonstrated a significant association between eating behaviours and energy intake. Furthermore, Pallister et al. [17] used a liking questionnaire in a UK twin cohort study to evaluate its usefulness to investigate the interaction between genetics and the liking of different fruits, vegetables, meats, and different tastes.

The liking of a specific food or set of foods primarily reflects the cultural environment in which an individual is brought up in and their individual experiences with such food [18]. However, the globalization of the food supply and the increase in disposable income has resulted in a diet where more products are derived from animals and energy dense sources, and the proliferation of Western-style highly palatable foods such as hamburgers, soft drinks, and pizza has created multiple problems in both developed and developing nations [19]. Such issues appear to be increasing in developing countries and include an increased prevalence of overweight and obese children and adolescents [20]. There appears to be a dramatic transition of food consumption patterns in a number of developing nations [21].

Exploring the relationship between the liking of food and dietary intake has not been widely studied; however, as indicated in the aforementioned studies, liking of a food appears to be one of the key factors influencing intake. To enable us to effectively use and interpret a FLQ it is first necessary to determine if a FLQ can appropriately measure food liking when compared to more established laboratory assessment methods in our two cultures population groups that have never been investigated. Therefore, the aim of this study is to compare a food liking questionnaire with food liking measured in a laboratory setting in a sample of Thai and Australian adults.

## 2. Materials and Methods

### 2.1. Participants

Seventy Australian and Thai non-smoking participants (35 Australian, 35 Thai, with a mean (SD) of 19.9 (3.0) years), 51% of which were male in both countries, were recruited to take part in the study. Australian participants were recruited from undergraduate courses at Deakin University, Melbourne, Australia using a range of strategies. Posters were distributed around campus and advertising material was distributed by study personnel to potential participants at locations around the campus. Presentations were also made to first-year undergraduate students enrolled in courses offered within the School of Exercise and Nutrition Sciences at Deakin University. Lastly, the study was advertised through social media. Thai participants were recruited via presentations to first-year undergraduate students enrolled in the Faculty of Science and Technology courses at Rajamangala University of Technology Tawan-ok, Thailand.

Participants were eligible if they were non-smokers, aged over 18 years, in good health, and had no allergies to any foods or food products, as determined through self-report using a short screening questionnaire prior to testing. Ethics approval was obtained from the Human Research Ethics Committee at Deakin University (HEAG-H 102_2016) and all participants who agreed to participate in the study provided written informed consent.

### 2.2. Procedure and Design

Data for the laboratory-based liking assessment were collected using the Compusense^®^ Cloud (Guelph, ON, Canada) for Australian samples. Hard copy paper questionnaires were used for the Thai sample. Participants were tested in individual booths with white lights and controlled air conditions in the sensory laboratories located at both the Centre for Advanced Sensory Science located within Deakin University and the Faculty of Science and Technology located within Rajamangala University of Technology. Participants were asked to refrain from eating, drinking (except room temperature water), brushing their teeth, and chewing gum for one hour prior to testing.

The participants first completed the food liking questionnaire (FLQ), then tasted and rated their liking of a selection of ten commercially available food items listed on the FLQ, see Table 1. The food items were selected to be representative of the commonly consumed foods within each food group. For example, for the FLQ group ‘soft drink’ orange Fanta was used in the laboratory testing, and for ‘potatoes chips’ Smith original chips were used for the Australian testing and Lay’s original for Thai. For both the questionnaire and laboratory-based liking assessment, liking was measured using a nine-point hedonic scale. This scale consists of a series of nine verbal categories representing degrees of liking from ‘dislike extremely’ to ‘like extremely’. For subsequent quantitative and statistical analysis, all verbal categories were converted to numerical values: ‘like extremely’ was coded as ‘9’, ‘dislike extremely’ as ‘1’. Participants rated their imagined (for FLQ) and experienced hedonic response for the food items on a nine-point hedonic scale. Both tasks were completed on the same day and all tests were repeated one week after the original session.

### 2.3. Food Liking Questionnaire

A modified version of a FLQ from Duffy et al. [11] was adapted for culturally relevant Australian and Thai foods. The Australian version of the questionnaire contained 73 food items, and the Thai questionnaire contained 89 food items. As many foods as possible were kept consistent between the Australian and Thai questionnaires to allow for a direct comparison. Examples of foods used in both questionnaires included: beef, cornflakes, potato chips, strawberries, pizza, and chocolate. The Australian questionnaire included the following culturally specific foods: Kentucky Fried Chicken (KFC) and rotisserie chicken. The Thai questionnaire included the following culturally specific foods: chilli dip, fermented fish, foods that have coconut milk/oil, spicy curry, Tom yum, sticky rice, Thai dessert made from egg yolk and sugar, fruit in thick syrup, and sweet test fruits. Both FLQs contained the instruction “*if you have never eaten a particular food, or never experienced one of the listed items, please rate the item as ‘neutral’*”. The Thai questionnaire was translated directly into Thai, see Table 2, by the lead author, a Thai researcher based in Australia, and was reviewed by one co-author, a Thai researcher based in Thailand, to ensure cultural appropriateness and accuracy.

### 2.4. Statistical Analyses

Statistical analyses were carried out using SPSS version 25.0 (IBM Corporation, Armonk, NY, USA). In order to detect a minimum of one unit mean difference on the nine-point hedonic scale between Australian and Thai samples with 80% power and a standard deviation of 1.5, a sample size of 35 per group was needed. Three different sets of Intra-Class Correlation Coefficients (ICC) were calculated. The first set of ICC was used to determine the comparability between test and re-test results of the FLQ. The second set of ICC was used to determine the comparability between test and re-test results of the laboratory-based liking assessment. The third set of ICC was used to determine the comparability between the results of the FLQ and the laboratory-based liking assessment. The ICC values were interpreted as poor (<0.40), moderate (0.40–0.59), good (0.60–0.74), and excellent (0.75–1.0) [22]. An independent sample *t*-test was used to compare the food liking groups between countries. A value of *p* < 0.05 was considered statistically significant.

## 3. Results

### 3.1. Test-Retest Food Liking Questionnaire and Laboratory-Based Liking Assessment Reliability

The level of ICC between test–retest of FLQ are reported in Table 3. Reliability for all the food items in FLQ was in the moderate range (0.40–0.59), except for broccoli which was in the excellent range (0.75–1.0).

The level of ICC between test–retest of the laboratory-based liking assessment, ranged from 0.55 to 0.85, as shown in Table 3. The degree of reliability was excellent (0.75–1.00) for instant vegetable soup and broccoli, good (0.60–0.74) for Tim Tam, orange Fanta, ice cream, apple puree, chilli sauce, and Tom yum soup, and moderate (0.40–0.59) for potato chips and butter.

### 3.2. Food Items Liking Comparability between the Questionnaire and Laboratory-Based Liking Assessment

The level of ICC between the FLQ and the representative food in the laboratory-based liking assessment ranged from 0.22 to 0.82, see Table 4. The degree of comparability was excellent (0.75–1.00) for broccoli; good (0.60–0.74) for potato chips and butter; and moderate (0.40–0.59) for sweet biscuits, soft drink, vegetable soup, and ice cream. The degree of comparability was poor (<0.40) for apple puree, chilli sauce, and Tom yum soup. When the analyses were repeated for men and women separately in Australian and Thai samples, the degree of comparability of FLQ and laboratory-based liking assessment was similar (data not shown).

### 3.3. Using the Food Liking Questionnaire to Discriminate between Thai and Australian Cultures

Independent sample *t*-tests were used to compare FLQ and a laboratory-based liking assessment between Australian and Thai samples, see Table 5. Statistically significant mean differences (*p* < 0.05) were observed in three food items: soft drink (mean difference (MD) −1.47 Australian vs. Thai), vegetable soup (MD = 1.58), and broccoli (MD = 1.36). Furthermore, the laboratory-based liking assessment found statistically significant differences in an additional four food items compared to the FLQ: Tim Tams (MD = 0.63), potato chips (MD = −0.542), apple puree (MD = 2.11), and chilli sauce (MD = 1.70).

## 4. Discussion

The objective of this study was to compare a FLQ with a laboratory-based liking assessment of ten representative foods in Australian and Thai settings, in order to determine whether the FLQ will be a suitable measurement tool in large-scale studies to compare food liking across cultures. The test–retest reliability of the FLQ and laboratory-based liking assessment were found to be moderate to excellent for both the FLQ and laboratory-based liking assessment, with an ICC range of 0.41–0.85 [22].

When comparing the FLQ with a laboratory-based liking assessment of individual food items, the comparability was moderate or high for seven food items (ICC range 0.43–0.82). However, comparability was poor for three food items: liking for apple measured on the FLQ compared with apple puree in the laboratory testing (ICC 0.22), the heat/burn of a spicy meal measured on the FLQ compared with chilli sauce (ICC 0.29), and Tom yum soup (ICC 0.31) in the laboratory taste testing. These comparability results may be explained by the differences in the food items assessed. The laboratory-based liking assessment asked subjects to rate their liking of a number of specific foods immediately after tasting. Conversely, the FLQ asked subjects to rate their liking of a number of foods without tasting the foods and this method may be more of a reflection of past experiences and memories of the food items [5,18]. Laboratory food testing provides a direct measure of liking of the food as consumed [13], with little influence of memory [5,18]. For example, a liking of apple may not equate to a liking of apple in puree form and a liking of the heat burn of a spicy meal, may not translate to a liking of chilli sauce eaten independently of the whole meal. These three food items deviated the most between the FLQ representative food and the actual food tasted in the laboratory and this may explain the poor comparability between methods. Taken as a whole, the results obtained indicate that while the FLQ appears an effective measurement tool to determine the liking across general food items in larger-scale studies, a laboratory-based sensory assessment may be necessary for measuring liking of the specific food products and laboratory testing will be required for direct product comparison. Similar results have been observed in previous studies comparing FLQ and a laboratory-based liking assessment. Deglaire et al. [23] reported on the reliability of utilising a food liking questionnaire to assess liking for salty, sweet, and fatty foods in a large population study. Deglaire observed that the questionnaire-based assessment of food liking was a robust method to collect liking data from large population studies. However, the authors noted that a liking value based on laboratory testing gives a direct measure of a liking value of the perceived flavour of the foods that are actually tasted, as opposed to a questionnaire where the liking value is based on the subject’s memories or experience. Cardello and Maller [13] noted that the liking response on the questionnaire was driven more on experience or memory of food, whereas the laboratory-based liking was based on the actual tasting of food samples.

The current results, combined with those of Duffy et al., Deglaire, and Cardello and Maller [11,12,13] indicate that for larger population studies, using a questionnaire to assess food liking is an appropriate data collection tool as it is reliable and is comparable with laboratory taste testing. In addition, the questionnaire has a significant benefit when assessing links of food liking with diet and anthropometry in that it has the potential to provide a representative view of an individual’s liking of a broad range of food groups, compared with what an individual may rate for a specific tasted food. This may provide a benefit when assessing links of food liking with diet and health indices.

One of the aims of our ongoing research program is to explore cultural differences in food liking in similarly aged sample populations. Therefore, we explored the ability of the FLQ to distinguish food liking between two sample populations. The FLQ was able to detect significant differences in food liking observed between Australian and Thai subjects using both FLQ and the laboratory-based sensory assessment. This indicates that the FLQ is able to discriminate between cultural differences in food liking, providing further confidence in its usefulness in exploring cultural differences in large studies. There was a significant difference in food liking identified in the FLQ and the laboratory-based liking assessment for three food items, including soft drink, vegetable soup, and broccoli. However, the laboratory testing found differences in an additional four food items compared to the FLQ. These items include Tim Tams, potato chips, apple puree, and chilli sauce. The differences in the liking of these four food items might be due to a greater familiarity with these foods within Australia and less familiarity within Thailand. For example, Thai participants’ liking ratings during the laboratory testing of Tim Tams and apple puree were significantly lower compared to the Australian subjects. This finding supports other research studies that show a lack of familiarity may influence the liking of a food [24,25,26,27,28].

The present study has a number of limitations that should be noted. The reliability between the FLQ and laboratory-based liking assessment was completed in participants from Australia and Thailand using foods specific to their culture. Therefore, the results may not be generalizable to other population groups. Further, only a representative number of food items were used in the laboratory-based liking assessment.

The present study used a relatively small sample size, which was based on an estimated difference of one unit on a nine-point hedonic scale. As such, the present study can be seen as the first step in our understanding of the relationship between the food liking questionnaire and direct sensory testing in a cross-cultural sample and sets the stage for larger studies. It is important to note that the present study did not aim to develop a method to replace the sensory evaluation of taste liking; rather, it aimed to propose an alternative method to obtain a general liking of food when an actual measurement is not possible.

## 5. Conclusions

The findings of this study demonstrated that the FLQ reflects the liking ratings in laboratory taste-testing and is an appropriate method to investigate food liking in large population groups including cross-cultural studies. The test-retest reliability of the FLQ and laboratory taste-testing were also assessed. This study concludes that the FLQ is also able to detect differences in liking between the Australian and Thai populations. Laboratory-based sensory testing remains the recommended method for direct product comparison.

## Figures and Tables

**Table 1:** Food items on the questionnaire and commercial foods.

Food Items on Questionnaire	Commercial Foods
Sweet biscuit	Chocolate biscuit (Arnott’s Tim Tam original)
Soft drink	Orange soft drink (The Coca-Cola Company)
Vegetable soup	Instant vegetable soup (Continental Homestyle vegetable)
Potato chips	Potato chips (for Australia: Smith; for Thai: Lay’s original)
Ice cream	Ice cream (for Australia: A2 milk classic vanilla ice cream; for Thai New Zealand Natural Premium Ice Cream classic vanilla ice cream)
Butter	Butter (Beautifully Butterfully butter, salt block)
Broccoli	Broccoli (boiled, fresh)
Apple	Apple puree (Sweet Valley)
The heat/burn of a spicy meal	Chilli sauce (Mars Food Australia hot chilli (under the MasterFoods brand)) and Tom yum soup (Roi Thai tomyum soup with coconut milk)

**Table 2:** The nine-point hedonic scale direct translation in to Thai.

English	dislike extremely	dislike very much	dislike moderately	dislike slightly	neither like or dislike	like slightly	like moderately	like very much	like extremely
Thai	ไม่ชอบ มากที่สุด	ไม่ชอบ มาก	ไม่ชอบ ปานกลาง	ไม่ชอบ เล็กน้อย	บอกไม่ได้ว่าชอบ หรือไม่ชอบ	ชอบ เล็กน้อย	ชอบ ปานกลาง	ชอบ มาก	ชอบ มากที่สุด

**Table 3:** Comparing mean score between test and re-test and reliability of Intra-Class Correlation Coefficients (ICC) of food liking questionnaire and laboratory-based liking assessment.

Food Items in FLQ	Test (*n* = 70)	Re-Test (*n* = 70)	95% CI of the Difference	ICC	Food Items in Sensory Laboratory Testing	Test (*n* = 70)	Re-test (*n* = 70)	95% CI of the Difference	ICC
	M ± SD	M ± SD				M ± SD	M ± SD		
Sweet biscuit	7.50 ± 1.47	7.64 ± 1.51	−0.47, 0.19	0.58	Tim Tam	8.01 ± 1.50	8.33 ± 1.05	−0.54, −0.09	0.73
Soft drink	6.30 ± 2.19	6.51 ± 2.01	−0.68, 0.25	0.57	Orange Fanta	7.14 ± 1.65	7.31 ± 1.46	−0.48, 0.14	0.66
Vegetable soup	6.41 ± 1.92	6.31 ± 2.00	−0.36, 0.56	0.51	Instant vegetable soup	5.24 ± 2.43	5.23 ± 2.30	−0.37, 0.40	0.77
Potato chip	7.44 ± 1.28	7.73 ± 1.28	−0.57, 0.00	0.56	Potato chips	7.83 ± 1.25	7.80 ± 1.17	−0.23, 0.29	0.59
Ice cream	8.16 ± 1.29	8.01 ± 1.17	−0.18, 0.46	0.41	Ice cream	8.06 ± 1.21	8.16 ± 1.15	−0.34, 0.14	0.64
Butter	6.39 ± 1.78	6.46 ± 1.93	−0.52, 0.38	0.48	Butter	5.99 ± 1.81	6.40 ± 1.84	−0.83, 0.00	0.55
Broccoli	6.60 ± 2.09	6.41 ± 2.37	−0.19, 0.56	0.75	Broccoli	5.93 ± 2.52	5.89 ± 2.51	−0.29, 0.37	0.85
Apple	7.56 ± 1.11	7.54 ± 1.00	−0.24, 0.27	0.50	Apple puree	5.27 ± 2.47	5.67 ± 2.43	−0.83, 0.03	0.72
The heat/burn of a spicy meal	6.61 ± 2.05	6.46 ± 2.10	−0.33, 0.64	0.52	Chilli sauce	3.79 ± 2.45	4.00 ± 2.32	−0.69, 0.26	0.65
					Tom yum soup	4.07 ± 2.32	4.20 ± 2.57	−0.58, 0.32	0.71

Note: Classification of ICC value: <0.40 = poor; 0.40–0.59 = moderate; 0.60–0.74 = good; 0.75–1= excellent. Standard Deviation (SD), Confident Interval (CI).

**Table 4:** Comparing mean score and reliability (ICC) between food liking questionnaire and laboratory-based liking assessment.

Food Items in FLQ	M ± SD (*n* = 70)	Food Items in Sensory Laboratory Testing	M ± SD (*n* = 70)	95% CI of the Difference	ICC
Sweet biscuit	7.57 ± 1.33	Tim Tam	8.17 ± 1.20	0.22, 0.61	0.43
Soft drink	6.41 ± 1.86	Orange Fanta	7.23 ± 1.42	0.41, 0.72	0.59
Vegetable soup	6.36 ± 1.70	Instant vegetable soup	5.24 ± 2.23	0.37, 0.70	0.56
Potato chip	7.59 ± 1.13	Potato chips	7.81 ± 1.08	0.59, 0.82	0.73
Ice cream	8.09 ± 1.04	Ice cream	8.11 ± 1.07	0.31, 0.66	0.51
Butter	6.42 ± 1.60	Butter	6.19 ± 1.61	0.46, 0.75	0.62
Broccoli	6.51 ± 2.09	Broccoli	5.91 ± 2.42	0.72, 0.88	0.82
Apple	7.55 ± 0.92	Apple puree	5.47 ± 2.28	−0.02, 0.43	0.22
The heat/burn of a spicy meal	6.54 ± 1.81	Chilli sauce	3.89 ± 2.17	0.06, 0.49	0.29
		Tom yum soup	4.14 ± 2.26	0.09, 0.51	0.31

Note: Classification of ICC value: <0.40 = poor; 0.40–0.59 = moderate; 0.60–0.74 = good; 0.75–1 = excellent.

**Table 5:** Comparison between the food liking questionnaire and laboratory-based liking assessment rating between Australian and Thai samples

Food Item in FLQ	Australian *n* = 35	Thai *n* = 35	95% CI of the Difference		Food Items in Sensory Laboratory Testing	Australian *n* = 35	Thai *n* = 35	95% CI of the Difference	
	M ± SD	M ± SD		*t*		M ± SD	M ± SD		*t*
Sweet biscuit	7.94 ± 0.82	7.20 ± 1.53	−0.50, 0.67	0.29 ^n.s.^	Tim Tam	8.48 ± 0.60	7.85 ± 1.53	0.07, 1.18	2.25 **
Soft drink	5.67 ± 1.74	7.14 ± 1.68	−2.29, −0.65	−3.58 **	Orange Fanta	6.58 ± 1.40	7.87 ± 1.12	−1.89, −0.67	−4.23 ***
Vegetable soup	7.15 ± 0.87	5.57 ± 1.95	0.86, 2.30	4.37 **	Instant vegetable soup	6.18 ± 1.37	4.28 ± 2.51	0.93, 2.86	3.926 ***
Potato chips	7.57 ± 1.23	7.60 ± 1.03	−0.57, 0.51	−0.10 ^n.s.^	Potato chips	7.54 ± 1.13	8.08 ± 0.96	−1.04, −0.04	−2.15 **
Ice cream	8.14 ± 0.80	8.02 ± 1.23	−0.38, 0.61	0.45 ^n.s.^	Ice cream	8.00 ± 1.07	8.21 ± 1.07	−0.72, 0.29	−0.83 ^n.s.^
Butter	6.42 ± 1.62	6.41 ± 1.59	−0.75, 0.78	0.03 ^n.s.^	Butter	6.50 ± 1.51	5.88 ± 1.66	−0.14, 1.37	1.61 ^n.s.^
Broccoli	7.18 ± 1.14	5.82 ± 2.57	0.40, 2.23	2.84 **	Broccoli	6.48 ± 1.73	5.32 ± 2.85	0.02, 2.28	2.04 *
Apple	7.65 ± 0.87	7.44 ± 0.96	−0.22, 0.65	0.97 ^n.s.^	Apple puree	6.52 ± 1.89	4.41 ± 2.15	1.14, 3.08	4.36 ***
The heat/burn of a spicy meal	6.52 ± 1.36	6.54 ± 2.19	−0.88, 0.85	−0.03 ^n.s.^	Chilli sauce	4.74 ± 2.22	3.04 ± 1.76	0.74, 2.65	3.54 ***
					Tom yum soup	4.44 ± 2.34	3.87 ± 2.17	1.14, 1.60	0.97 ^n.s.^

Note: Definitions—Standard Deviation (SD), mean (M), number of participants in each group (*n*). * *p* values are for the comparison food liking rating between Australian and Thai samples were determined using independent sample *t*-tests. Significance indicated the * *p* < 0.05, ** *p* < 0.01, *** *p* < 0.001, and n.s. indicates not significant at *p >* 0.05.
